# On the Nexus of the Spatial Dynamics of Global Urbanization and the Age of the City

**DOI:** 10.1371/journal.pone.0160471

**Published:** 2016-08-04

**Authors:** Sebastian Scheuer, Dagmar Haase, Martin Volk

**Affiliations:** 1 Landscape Ecology Lab, Geography Department, Humboldt-Universität zu Berlin, Berlin, Berlin, Germany; 2 Department of Computational Landscape Ecology, Helmholtz Centre for Environmental Research–UFZ, Leipzig, Saxony, Germany; Universite de Namur, BELGIUM

## Abstract

A number of concepts exist regarding how urbanization can be described as a process. Understanding this process that affects billions of people and its future development in a spatial manner is imperative to address related issues such as human quality of life. In the focus of spatially explicit studies on urbanization is typically a city, a particular urban region, an agglomeration. However, gaps remain in spatially explicit global models. This paper addresses that issue by examining the spatial dynamics of urban areas over time, for a full coverage of the world. The presented model identifies past, present and potential future hotspots of urbanization as a function of an urban area's spatial variation and age, whose relation could be depicted both as a proxy and as a path of urban development.

## Introduction

Urbanization is an increasingly dominant, although heterogeneous, process of human behavior and settling on Earth, and a key driver of global ecological change. Cities are hotspots of demographic and economic development, generating more than 90% of the global gross value added [1]. The global urban population is increasing dramatically, from 13% in 1900 to an estimated 70% in 2050 [2].

**Fig 1:**
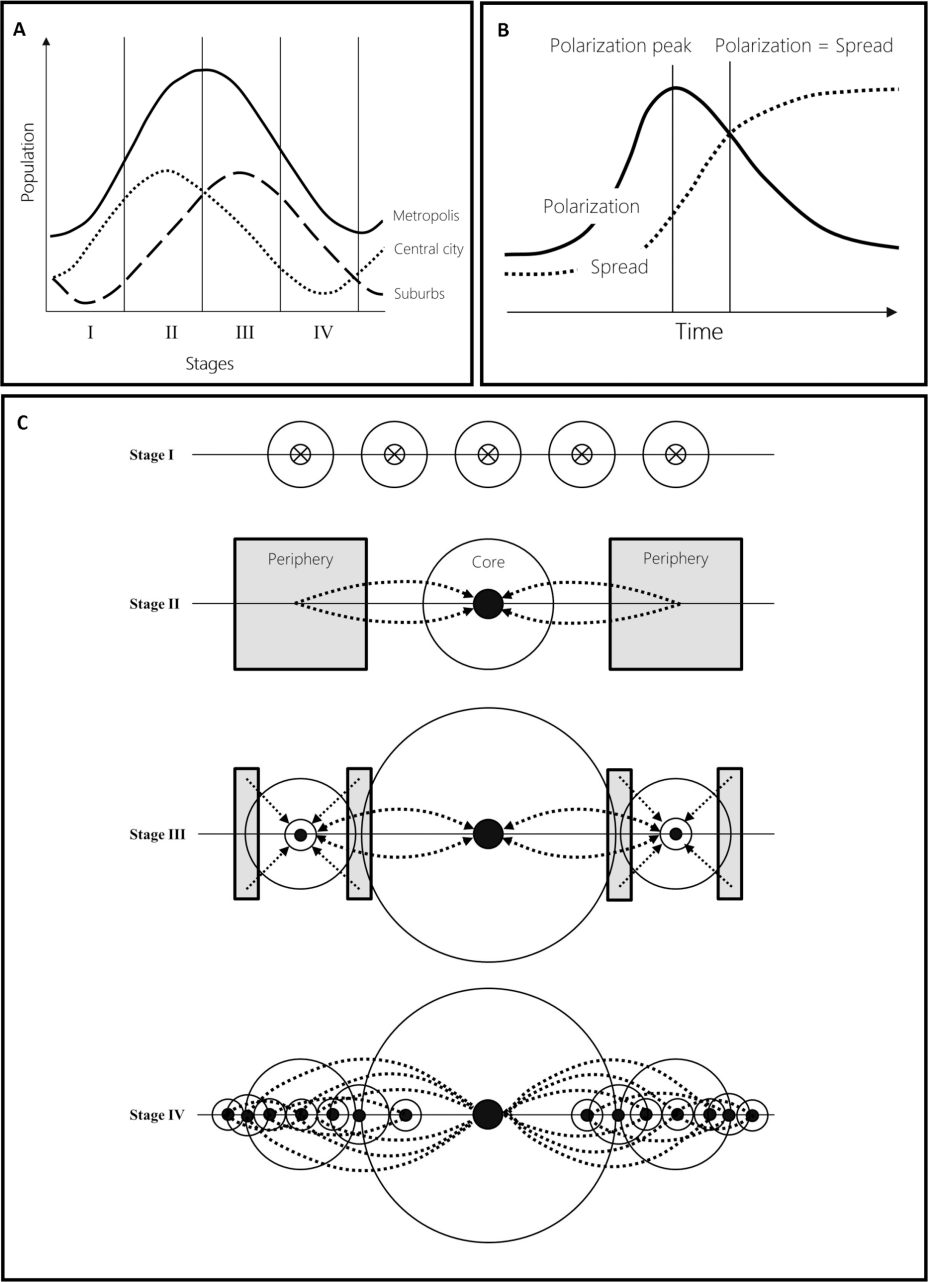
Conceptual models of urban development. (A) Stages of urban development model proposed by van den Berg [15], with the four stages urbanization, suburbanization, desurbanization, and re-urbanization. The stages are distinguished on the basis of demographic dynamics. (B) The polarization and spread model of urban development devised by Myrdal [16]. Essentially, the model is focused on the accumulation of urban activities and assets, and their subsequent spread, i.e., dispersal, into the urban hinterland. (C) The core-periphery model by Friedmann that conceptualizes the transformation of functionally rather isolated, pre-industrial cities into interdependent, multi-centric urban agglomerations [20]. Stage I corresponds to pre-industrial conditions that are represented by independent core cities lacking hierarchical relations. Industrialization accompanied by an accumulation of activities and resources effectively leads to the development of dominant urban cores surrounded by peripheral, rather rural areas (stage II). Exchange and dispersal of urban and industrial activities into the peripheries of urban core cities leads to a functionally more interdependent system of hierarchically organized cities and a suburban space (stage III). This development eventually results in a post-industrial, functionally fully interdependent urban system (stage IV).

There are many ways to approach the process of urbanization as such: The historical perspective [3], the spatial perspective [4, 5], the network and power hierarchies perspective [6], the land use perspective [7] or the sustainability and lifestyle debate [8]. Spatiotemporal typologies of urbanization and the dynamics of its change have been studied intensely by geographers, economists and also social scientists for many decades [9]. The reasoning on the drivers that are behind the aforementioned processes and types of urbanization vary between the economic competition between different land use(r)s [10, 11] or between social/ethnic groups [12–14]. Other models employ the changing concentration of population in an urban region/agglomeration as key by describing urbanization on the basis of population, population density or labor, hence necessitating reliable socio-demographic data. Among these models are the stages of urban development model (Fig 1A) by van den Berg [15] and the polarization and spread model (Fig 1B) proposed by Myrdal [16]. The van den Berg model captures growth and shrinkage processes of core cities and their periphery as a function of relative and absolute population change in the agglomeration. It distinguishes between the stages of (i) urbanization, i.e., absolute population growth in the core city and thus the agglomeration as a whole; (ii) suburbanization, i.e., absolute population growth in the agglomeration, with growth being relatively concentrated on the suburban space. The core city stagnates; (iii) desurbanization, i.e., absolute population decline in the core city, its periphery and thus the urban agglomeration in total; and more recently (iv) re-urbanization, i.e., the recurring population growth in the agglomeration’s core city [17, 18]. The polarization model by Myrdal describes urbanization as the result of the processes of (i) polarization, i.e., a concentration or accumulation of activities, resources, and wealth, leading to the emergence of core cities over time; and (ii) spread, i.e., the subsequent dispersal of these activities and assets into the urban periphery and hinterland. (Fig 1B) [16].

On a global scale, these processes of urbanization pose a significant impact. Today's urban areas are estimated to comprise approximately 5% of the global land surface [5, 7]. This area is expected to increase by 200% until 2030 [1], further underscoring the concentration of people and activities within a small part of the available land surface. The majority of growth is assumed to occur in small- to medium-sized cities in African and Asian developing countries and in Latin America; the lowest expansion rates are expected for Europe, North America, and Australia/Oceania [1, 2, 19].

Despite these spatial effects, the conceptual models of urban development as shown in Fig 1A and Fig 1B ignore this important spatial dimension. This behavior poses challenges to the understanding of most related, important issues such as humans’ quality of life, food and energy consumption, health, climate and hunger vulnerabilities and risks, which urgently require knowledge regarding the spatial dimension of urban growth. Consequently, spatially explicit approaches to urbanization have been conceptualized to describe spatial urban patterns. An example of such models is the core-periphery model by Friedmann [20] that describes the transition of a pre-industrial system of independent urban cores (Fig 1C, stage I) to a post-industrial, functionally interdependent and hierarchical urban system (Fig 1C, stage IV).

Other models on the dynamics and transformation of urban development are based on the chaos theory [21], the theorem of fractal development represented by means of cellular automata [22, 23] or systemic self-organization [24]. Many, often remote sensing-based studies seek to quantify the spatial effects of urban development by means of land-use change statistics, transition matrices and landscape metrics to uncover urban growth or shrinkage [25–28]. The implications and effects of urbanization on ecosystems, heat islands and natural capital are also studied extensively [29, 30]. Recent studies additionally focus on transforming qualitative and conceptual descriptions of urban systems into quantitative, spatially explicit definitions [31] and seek to identify novel means to describe the complex morphology of cities or urban systems [32].

Why do we develop another model of urbanization? Arguably, socio-demographic statistics are often coarse and not spatially explicit, thus making it difficult to implement conceptual urbanization models. Examining the expected future hotspots of urbanization, Africa in particular, this hindrance is further complicated by the fact that required data are often unreliable or wholly unavailable. In addition, the cadastral land register or land-use data are often not available in developing countries or are inconsistent over space and time, thereby necessitating laborious data integration. Although remote sensing data are globally available, they lack multi-temporal components [31], thus hampering the determination of urban history, i.e., the identification of the former and potentially ongoing phases of urbanization. However, remote sensing-based studies are also typically on a regional scale, thereby lacking in providing a global view of an increasingly urban world to better understand the future of urbanization. These limitations make it necessary at best to provide stakeholders and decision-makers with the tools needed to identify and manage urbanization and related problems on different spatial scales. To overcome some of the aforementioned limitations, we propose a global model of urban development that integrates conceptual views on urbanization into a multi-temporal spatial context. The new model develops statistical relations between the age of an urban area and its spatial dynamics over time. At least for the US, studies have found significant correlations between an urban agglomeration's age and the population size as well as population growth rates [33, 34], thus rendering age an important key variable when examining the urbanization process.

The remainder of this paper seeks to deepen the understanding of the relationship between urban area age and the spatial dynamics of the urban space over time. In order to obtain a more comprehensive view on urbanization as a process, additional variables—e.g., urban population count—will also be employed. Based on the identified linkages, a spatially explicit model of urbanization will be elicited.

The corresponding analysis is carried out in several stages. In a first step, general patterns of the model’s core variables urban area age and their spatial dynamics will be identified on a global level, upon which the basic assumptions of the proposed model will be formulated. The underlying data will be introduced accordingly. In a second step, the postulated assumptions, as well as the fundamental underlying structure of the variable’s relationships, will be analyzed and confirmed by means of a principal component analysis (PCA). The relationship between the model’s variables is then further explored by investigating selected case cities. In doing so, distinctive patterns of age and spatial dynamics are linked to specific processes of urbanization, and likewise states or “conditions” of urban developments. Building upon this knowledge, and by means of a bivariate local Moran’s I analysis, the proposed spatially explicit model of urbanization will be elicited thereafter. The paper closes with discussion and conclusions.

## Materials and Methods

**Fig 2:**
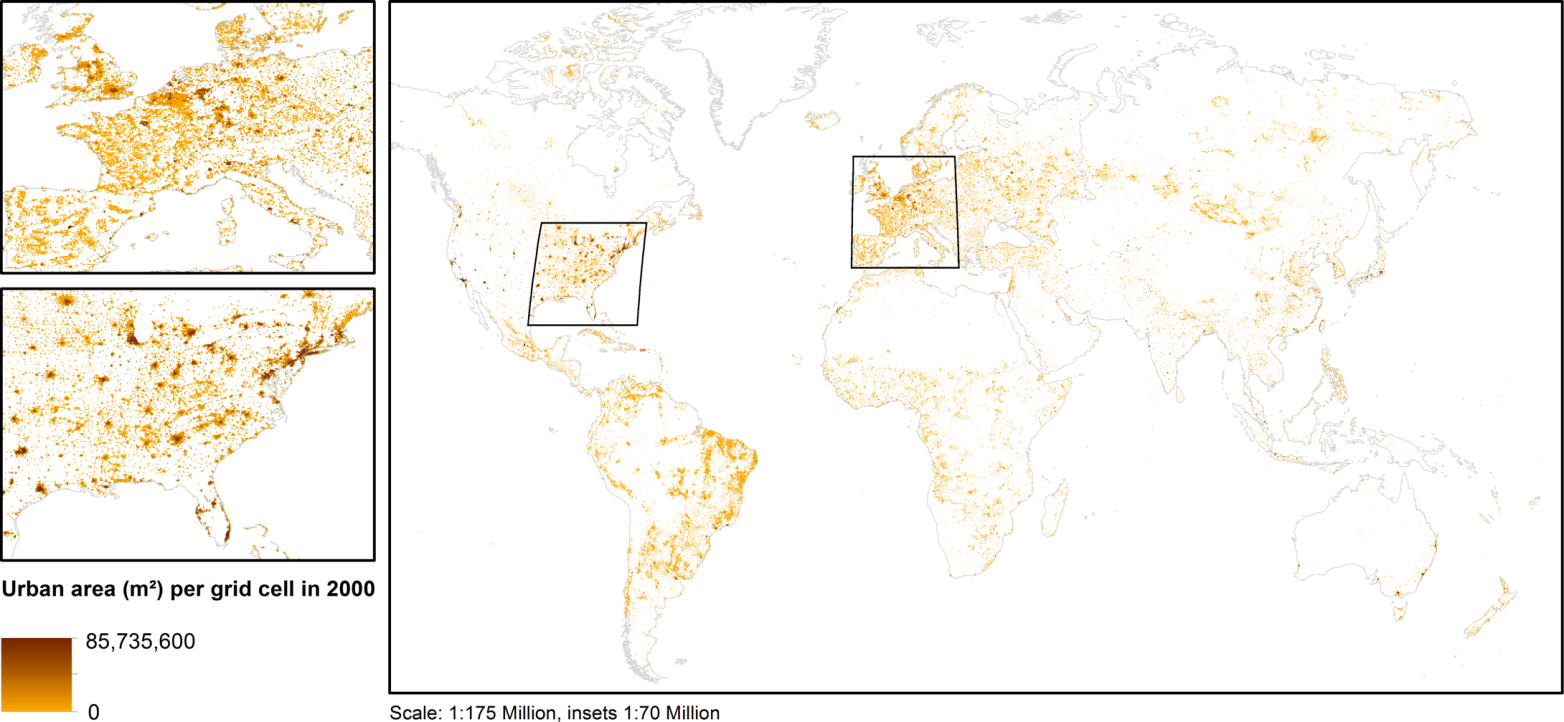
Total urban area (m^2^) per grid cell *uopp* for the year 2000 taken from the HYDE 3.1 database. Looking at the scale of the dataset, it becomes clear that *uopp*, i.e., the total urban area per grid cell (m^**2**^) is not necessarily corresponding to distinct cities, but encompasses the total urban space in the covered area. This might correspond to a subarea of a given city, e.g., in the case of megacities, to one or more (typically smaller) cities, as well as the suburban land or hinterland.

To obtain a long-term view on global urbanization, seven time steps have been included in the analysis: 1900, 1920, 1940, 1950, 1960, 1980, and 2000. The period chosen, from 1900 to 2000, is explained by the fact that modern urbanization as a dominant process in global land (use) development went together with industrialization, first in Europe and the US, starting after 1860…1890. The base data have been taken from the HYDE 3.1 database of Klein Goldewijk et al., which provides a global coverage of longitudinal land-use figures—i.e., cropland, pasture and built-up, urban area (Fig 2)—as well as urban and rural population counts for a 5’ x 5’ grid resolution [35, 36]. In HYDE 3.1, land-use is estimated for the complete Holocene, i.e., from 10,000 BC to 2000 AD, using time-dependent allocation algorithms. Such estimates are frequently associated with uncertainties. Klein Goldewijk et al. recognize uncertainties for their allocation model especially for earlier forecasts, and assume uncertainties in urban population counts between 1–5% for estimates between 1900 and 2000. Estimates for urban area, at least for the year 2000, as shown in Fig 2, are described as reasonably in line with third-party predictions [36]. Despite these uncertainties, the HYDE 3.1 database has been chosen as a base data provider since it ensures an internally consistent dataset.

**Fig 3:**
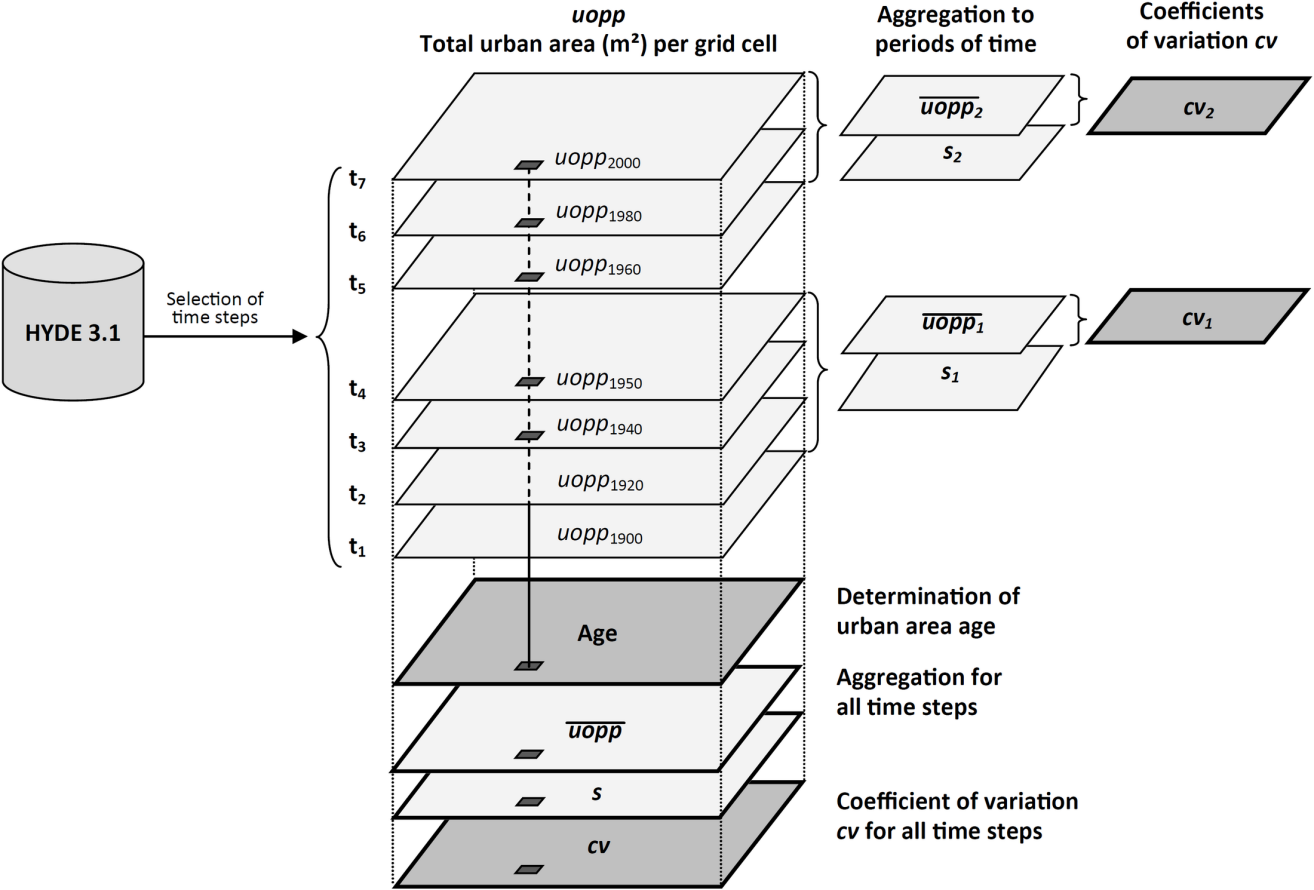
Data preparation to determine the age and relative spatial variability of urban area. The variable *uopp*—i.e., the total urban area (m^**2**^) per grid cell—has been taken from the HYDE 3.1 database for seven years (time steps), 1900, 1920, 1940, 1950, 1960, 1980, and 2000. The gridded *uopp* data have been overlaid to determine the age of an urban area. This has been done by counting the number of times a given grid cell features allocated urban land, where the first appearance of urban structures in a given grid cell is used as a proxy for age. Furthermore, the mean urban area (m^**2**^) per grid cell $\bar{uopp}$ has been determined as well as its standard deviation *s*, and subsequently, the coefficient of variation of urban area extent *cv* (in %) has been calculated with $cv=100(s∙{\bar{uopp}}^{-1})$ as a proxy of the spatial dynamics of urban area extent over time. Additionally, two subsets have been assessed that represent the periods 1900–1950 and 1960–2000.

Using the aforementioned time steps, the age of urban areas and their spatial dynamics over time have been determined (Fig 3). The analysis is based on a grid of constant resolution of 10’ x 10’ arc length; the slightly lower resolution compared to the original HYDE 3.1 database has been chosen due to technical reasons. These include limitations in the maximum size of a shapefile, which was exceeded using the original resolution, as well as considerations for further use of the data in other software packages for further analysis, as described later.

The spatial dynamics over time has been assessed by observing changes in the total size of an urban area (m^**2**^) per grid cell, in the following *uopp*, over the selected time steps, and is expressed as the relative variability of urban area extent, i.e., the coefficient of variation (in %), in the following *cv*, which has been found to be a suitable indicator to characterize the spatial patterns of urbanization over time [31]. Moreover, it has been emphasized that the analysis is based on a gridded data source with a constant resolution of 10’ x 10‘. This results in a varying absolute area per grid cell as a function of latitude. Hence, the use of a relative measure of variability such as *cv* is further indicated in order to be able to make meaningful comparisons between different grid cells: The higher *cv*, the higher the standard deviation of urban area in relation to its mean size over time, independent of the absolute magnitude of mean area or change. Thus, the higher *cv*, the higher the spatial impact of urbanization processes in relation to the previously present urban land. Consequently, making use of a relative measure of variability also adjusts for different city sizes in terms of their absolute spatial extent.

The age of urban land has been derived in an indirect manner. Since the urban area in a given grid cell may encompass several cities (Fig 2), it is not feasible to derive age based upon individual foundation dates per city. The earliest observation of urban land in a particular grid cell has been determined instead. This has been done by overlaying all time steps and counting the number of occurrences of urban structures per grid cell (Fig 3). E.g., if a grid cell features urban land in all seven time steps, the corresponding built-up land is assumed to have been founded in 1900 or earlier. If, on the contrary, a given grid cell shows urban land only in the most recent two time step 1980 and 2000, the age of the corresponding built-up area is deduced accordingly (S1 Table). Consequently, it is thus assumed that the higher the observation count, the older the corresponding urban space.

This method of estimation is characterized by some degree of uncertainty. It particularly relies on a “chronological order” of observations, i.e., an assumption of “spatiotemporal continuity”. A violation of this assumption results in observation counts not being interpretable as age. For the majority of all cases, 91.5% of total grid cells, this assumption holds true. Looking at the distinct counts of observations of urban land, no less than 73.4% follow the expected “chronological order” (S1 Table).

### Global Patterns of Age and Spatial Dynamics of Urban Land Area

**Fig 4:**
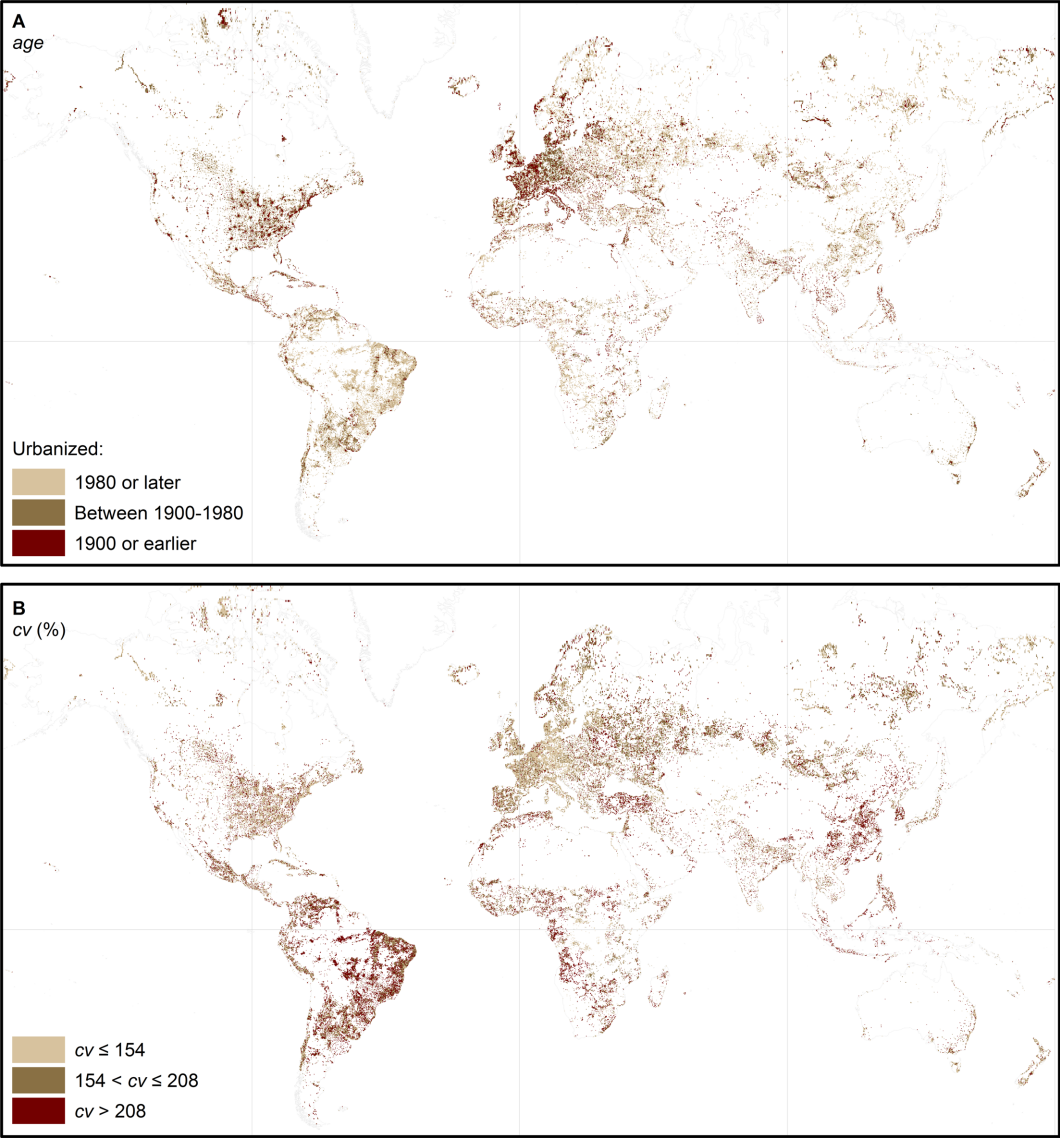
Global patterns of urbanization. (A) Age of an urban area in terms of the estimated year of its foundation. (B) Relative variability of the urban area extent expressed by the coefficient of variation *cv* (%). The classes of *cv* correspond to terciles. I.e., one-third of grid cells have a relative spatial variability lower than or equal to 154%; one-third have a relative spatial variability higher than 154%, but lower than or equal to 208%; and one-third have a relative spatial variability greater than 208%.

The resulting patterns for the two variables *cv* and age are visualized in Fig 4. At first sight, known patterns of urbanization become visible, e.g., relatively old European or North American built-up urban area dating back to 1900 and earlier. However, it also becomes clear that recent, i.e., younger, and more dynamic hotspots of urbanization are located primarily in the "Global South", i.e., in Latin America, South America, Africa, and Asia. In those areas, *cv* is relatively high compared to the north-western urban core areas. This observed pattern leads to the following fundamental assumptions: (i) Older urban areas are comparatively mature, i.e., "stable" in their spatial extent over time, and consequently, the relative variability *cv* is comparatively low; and (ii) recent urban developments, which could be indicative of on-going urbanization, suburbanization, or spread, are characterized by a higher relative variability of the urban area extent *cv*.

As described in Fig 3, the relative variability of the urban area extent has additionally been determined for two observation periods: 1900–1950 (*cv*_1_) and post-1950 (*cv*_2_). These periods were selected to distinguish earlier urbanization, systematic urban expansions of the late 19^th^ and early 20^th^ century and the interwar period in particular, from later stages, e.g., 1960s suburbanization.

A principal component analysis (PCA) was carried out using the software package SPSS version 22 by IBM to further explore the internal structure of the model variables. The PCA shall help to uncover the relationships between the model's core variables, age and *cv*. Total urban population count, a typical socio-demographic indicator of urbanization employed e.g. in the stages of urban development model shown in Fig 1A, and the total size of urban land have additionally been included in the PCA. Both variables have also been taken from the HYDE 3.1 database. In the PCA analysis, the Kaiser-Meyer-Olkin measure was 0.683, being classified as mediocre [37], with the Bartlett's Test of Sphericity being highly significant (*p* < 0.001). Thus, following the Kaiser criterion [38], two components were extracted with an eigenvalue greater than one. This two-component solution explains 84% of variance (S2 Table).

To aid interpretability, an orthogonal Varimax rotation was employed. The rotated solution reveals that the variables age, *cv*, *cv*_1_ and *cv*_2_ load on the first component, which explains 50.8% of variance (S2 Table). A first dimension of urbanization can thus be conceptualized as the spatiotemporal dynamics of built-up area. The PCA also confirms the aforementioned hypotheses that younger urban areas exhibit greater spatial dynamics, and vice versa (S1 Fig). Urban population count and total size of urban land both load positively on the second component, explaining 33.2% of variance. The coefficients of these two variables imply that the total size of urban land increases with a growing urban population (S3 Table), which is in accordance to previous findings [33, 34]. Hence, the second dimension of urbanization can be conceptualized as being related to socio-demographic pressures.

### Pathways of urban development

Developing the abovementioned assumptions, the relationship between the relative variability of urban area extent and its age should be explored further. In the remainder of the paper, the focus is thus placed on the first dimension of urbanization.

**Fig 5:**
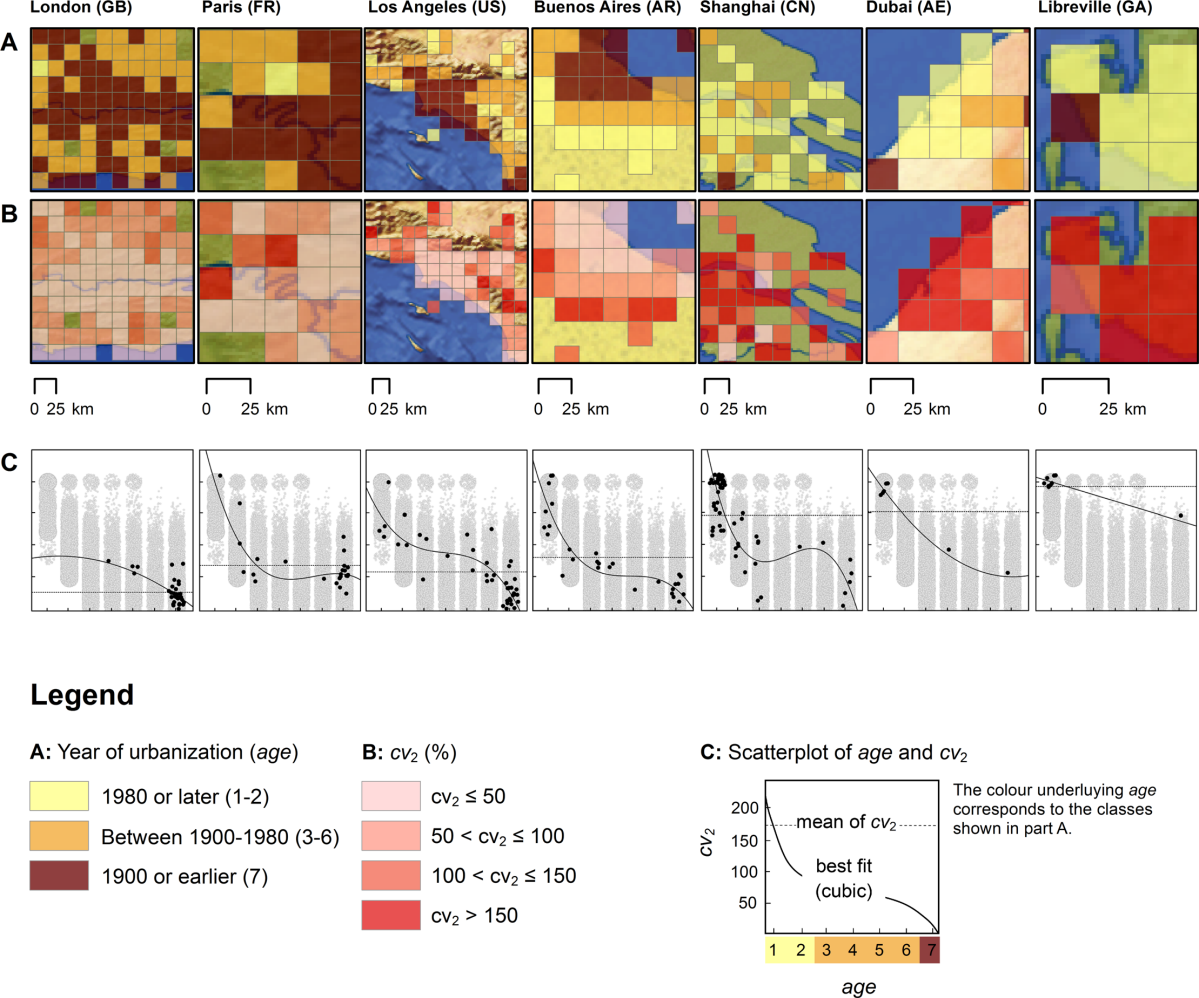
Pathways of urban development for selected case cities (urban regions). The figure visualizes the age of built-up, urban land and the relative variability of its extent both spatially and as a scatterplot. (A) Age of urban area. Older areas appear darker, younger areas appear brighter. (B) Relative variability of urban area extent 1950–2000, as measured by the variable *cv*_2_ (classification based on equal interval). By putting age and relative variability into context, it becomes clear that older areas tend to have lower values for *cv*_2_, indicating a lack of larger-scale spatial development in the study period. This is in line with the model’s assumptions. (C) The scatterplots visualize age (abscissa) and *cv*_2_ (ordinate) to ease the interpretation of A and B (please refer to the legend for axis properties). Each data point of the scatterplot corresponds to a grid cell. Grey data points in the background correspond to the complete dataset, black data points correspond to the grid cells of each case studied. By looking at these plots it becomes clear that recent, large-scale city expansions (e.g., in Shanghai or Dubai) are characterized by low age and a very high relative variability of spatial extent. On the contrary, old core cities (e.g., London) feature comparatively low values for *cv*_2_ and have a higher age. A best-fit polynomial model, plotted as a solid line, visualizes this observation for each case city. Furthermore, for each city, the mean value for *cv*_2_ is plotted as a dashed line.

Initially, the relation between the aforementioned variables will be studied for selected case cities—including their periphery and hinterland to some extent—to identify typical types or pathways of urban development (Fig 5). These cases include London (GB), Paris (FR), Los Angeles (US), Buenos Aires (AR), Shanghai (CN), Dubai (AE), and Libreville (GA). The choice of this sample was driven by the following criteria: (i) to achieve a broad selection of cases across continents; (ii) to cover the whole time period from 1900 to 2000 in terms of the year of city formation; and (iii) to study well-known cities with a complex history as well as lesser known cities in order to ensure that the model we develop is overall applicable and valid.

Distinct stages of urbanization, as exemplified by the known history of these cities, will be related to these pathways. However, instead of *cv*, the variable *cv*_2_ will be used in the remainder of this paper to quantify the relative variability of urban area extent because it emphasizes recent urban developments and better contrasts those with earlier ones, which we believe is of most relevance to decision-makers and stakeholders. As described above, the variable *cv*_2_ essentially captures the relative variability of urban area extent post-1950; focusing on changes of the extent of urban space in this period will thus reveal (i) newly built-up urban land since 1950, as a result of e.g. urban spread and suburbanization; and (ii) changes of spatial extent after 1950 to urban land built-up before 1950, e.g., as a result of systematic expansions of core cities, or re-urbanization and redensification. Finally, by relying on this more recent study period, uncertainty related to the spatial modelling of urban area is sought to be reduced [36].

The development of an urban area seems dependent on economic and demographic pressure, legislative boundaries and opportunities and obstacles for its spatial development [39]. London (GB), as a first example, experienced massive growth and expansion from the mid-19^th^ century industrialization period until the interwar period. Then, following the establishment of an urban green belt in the 1930s, London's spatial sprawl has mostly been halted [40]. Consequently, in particular the core city of London, being founded well earlier than 1900, is characterized by high age (Fig 5A). It further becomes clear that (macro-scale) extensions to this core city in the study period, 1950–2000, have been sparse, which is illustrated by the comparatively low relative variability shown in Fig 5B. The scatterplot visualizes this relationship (Fig 5C). Paris (FR) is the dominant French urban agglomeration. From 1801 until about the 1960s, its growth rate was approximately double that of any other major French city [41]. Major inner-city reconstructions were already undertaken at the end of the 19^th^ and early 20^th^ century by Haussmann. Restructuration and intra-urban redevelopments also followed the second world war, particularly the construction of La Défense and in the periphery, where large-scale, planned city extensions in the form of suburban housing complexes, the “grands ensembles”, were erected [41]. Los Angeles (US) is a typical example of 20^th^ century North-American suburbanization [34]. Buenos Aires (AR) exemplifies a colonially founded Latin American city, with suburbanization starting in the 1960s. Shanghai (CN) shows that a phase of relatively slow expansion in the 1970s and 1980s preceded a surge in growth following economic reforms in 1992, which lasts to today [42]. Dubai (AE) is another example of recent urbanization. Being a port town at the end of the 19^th^ century, growth and expansion of the city increased drastically following the discovery of oil in the 1960s and was further accelerated in the late 1990s. Finally, Libreville (GA), the capital of Gabon, is an example of African post-colonial city development. From Gabon's independence in 1960 until 2010, the city's population has increased by a factor of 22 [43].

Looking at these different cases, the following observations can be made: (i) in tendency, for a given city, older parts seem to feature comparatively lower coefficients of variation *cv*_2_; and (ii) newer urban development seems to be characterized by comparatively high coefficients of variation *cv*_2_. Both observations are reflected by the best-fit models shown in Fig 1C. However, there is evidence of substantial variation in *cv*_2_ not only between cities, but also within (higher) age classes of a given city. This variation seems to be the result of inner-city expansions, redevelopments, and potentially redensification.

### Identification and interpretation of patterns of *cv*_2_ and age

In the previous section, exemplary patterns of *cv*_2_ and age have been shown for selected case cities/urban areas, and first links between these patterns and various processes and “states” of urbanization—e.g., on-going and recent urban growth, suburbanization, as well as the emergence of core cities as a result of long-lasting polarization—have been established. To further explore the relation between both variables, additionally focusing on their spatial patterns to establish a spatially explicit view, a bivariate Local Moran's I statistics was applied. Bivariate Local Moran's I is an extension of the univariate Local Moran's I spatial autocorrelation statistics, which is available in the GeoDa tool [44, 45].

**Fig 6:**
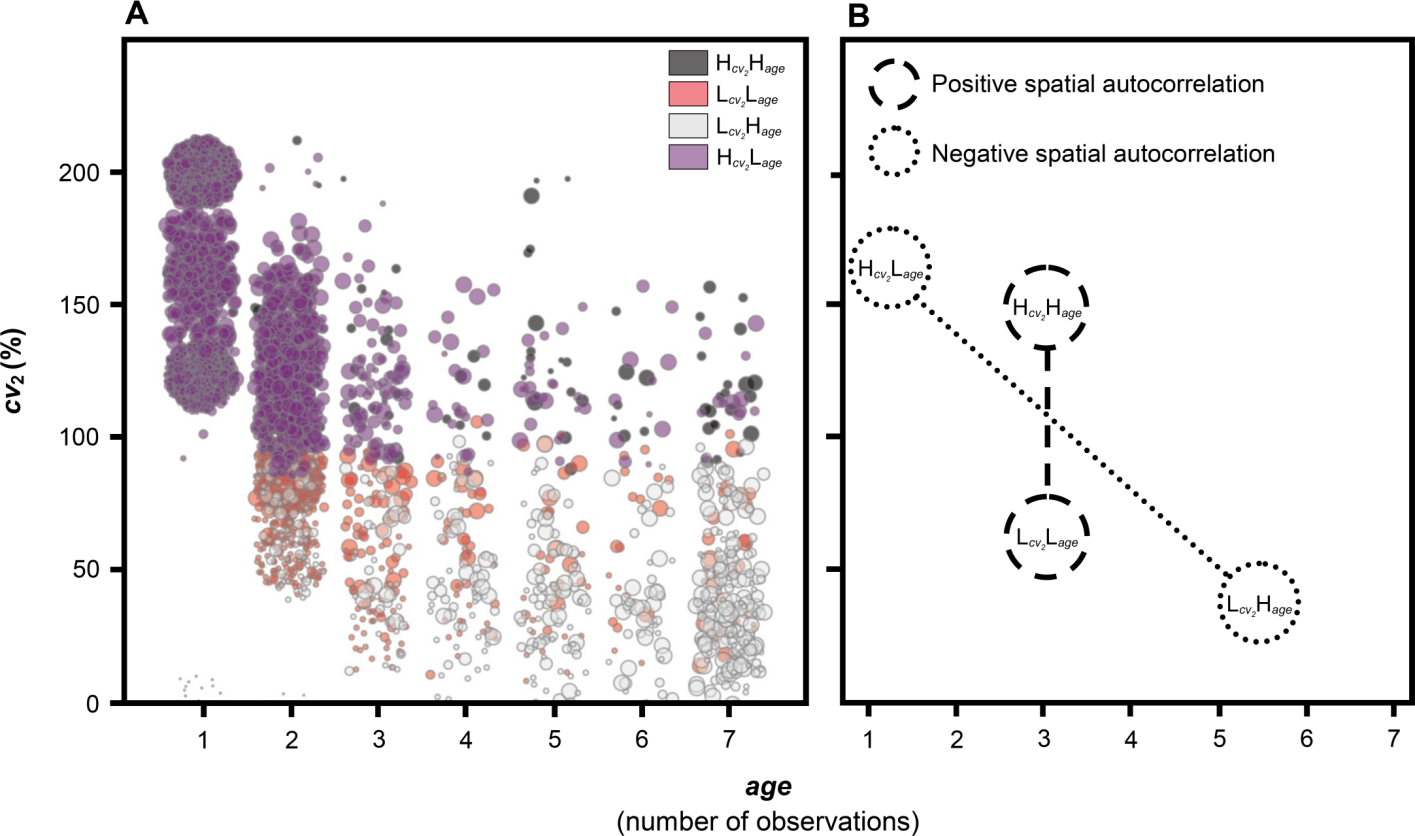
Bivariate Local Moran's I spatial autocorrelation analysis and hypothesized facets or pathways of urban development. (A) The scatterplot visualizes the relation between the age of an urban space and its mean relative spatial variability *cv*_2_. Each data point corresponds to a grid cell of the dataset. The color coding of each data point indicates the corresponding LISA cluster: $H_{{cv}_{2}}H_{age}$, High *cv*_2_-high age; $L_{{cv}_{2}}L_{age}$, Low *cv*_2_-low age; $L_{{cv}_{2}}H_{age}$, Low *cv*_2_-high age; $H_{{cv}_{2}}L_{age}$, High *cv*_2_-low age. (B) Schematic illustration of cluster mean values with a hypothetical pathway of urban development as a result of urbanization in terms of polarization (negative spatial autocorrelation) and an axis indicating processes of urban spread (positive spatial autocorrelation).

In doing so, statistically significant “combinations” of *cv*_*2*_ and age have been elicited, taking into account their spatial relationship. Furthermore, the identified patterns are explained conceptually, taking into consideration the processes of urban development. Local Moran's I identifies four clusters of spatial autocorrelation (LISA clusters): (i) High-high, i.e., high coefficient of variation *cv*_2_-high age (in the following $H_{{cv}_{2}}H_{age}$); (ii) Low-low, i.e., low coefficient of variation *cv*_2_-low age (in the following $L_{{cv}_{2}}L_{age}$); (iii) Low-high, i.e., low coefficient of variation *cv*_2_-high age (in the following $L_{{cv}_{2}}H_{age}$); and (iv) High-low, i.e., high coefficient of variation *cv*_2_-low age (in the following $H_{{cv}_{2}}L_{age}$). The former two clusters represent positive spatial autocorrelation, and the latter two, negative spatial autocorrelation (Fig 6).

Examining Fig 6, the clusters of negative spatial autocorrelation correspond to two opposing ends in the spectrum of age and *cv*_2_, which are thought to be linked to the process of polarization. $H_{{cv}_{2}}L_{age}$ denotes urban area of high relative spatial variability and low age, e.g., including (parts of) Dubai (AE), Libreville (GA), Shanghai (CN), Chengdu (CN) or New Delhi (IN). Consequently, it is assumed that the $H_{{cv}_{2}}L_{age}$ cluster marks recent, on-going processes of urbanization, i.e., current urban hotspots, as a result of polarization. This is supported by the fact that, compared with all other LISA clusters, $H_{{cv}_{2}}L_{age}$ has the significantly highest mean relative spatial variability, which is a strong indication for larger-scale transformations of land into urban space in relation to the present mean urban area. The $L_{{cv}_{2}}H_{age}$ cluster, conversely, typifies urban space of comparatively low relative variability of spatial extent and high age. For example, the core cities of London (GB), Paris (FR), Buenos Aires (AR), Boston (US), Cairo (EG), Manchester (GB), New York (US), St. Petersburg (RU) and Vienna (AT) fall into this cluster. It is thus assumed that the $L_{{cv}_{2}}H_{age}$ cluster indicates stable, “matured” urban cores, i.e., core cities lacking macro-scale spatial dynamics, which emerge due to long-lasting polarization processes. The $L_{{cv}_{2}}H_{age}$ cluster has the significantly lowest mean relative spatial variability (Table 1).

**Table 1:** Pairwise comparison of LISA cluster mean rank of relative spatial variability *cv*_2_.

	$H_{{cv}_{2}}H_{age}$	$L_{{cv}_{2}}L_{age}$	$L_{{cv}_{2}}H_{age}$
$L_{{cv}_{2}}L_{age}$	↓^a^		
$L_{{cv}_{2}}H_{age}$	↓	↓	
$H_{{cv}_{2}}L_{age}$	↑	↑	↑

$H_{{cv}_{2}}H_{age}$, High-high cluster; $L_{{cv}_{2}}L_{age}$, Low-low cluster; $L_{{cv}_{2}}H_{age}$, Low-high cluster; $H_{{cv}_{2}}L_{age}$, High-low cluster; ↑, mean rank significantly higher; ↓, mean rank significantly lower.

^a^ The table is read line-by-line. For example, the low-low cluster ($L_{{cv}_{2}}L_{age}$,) has a significantly lower mean rank of relative variability of urban area extent *cv*_2_ than the high-high cluster ($H_{{cv}_{2}}H_{age}$).

The clusters of positive spatial autocorrelation, i.e., $L_{{cv}_{2}}L_{age}$ and $H_{{cv}_{2}}H_{age}$ (Fig 6), are believed to represent facets of urbanization linked to urban spread. $L_{{cv}_{2}}L_{age}$ exemplifies urban areas of low to intermediate age in conjunction with low to intermediate mean relative spatial variability. The mean *cv*_2_ is significantly lower than for $H_{{cv}_{2}}L_{age}$, but also significantly higher than for $L_{{cv}_{2}}H_{age}$ (Table 1). This behavior is viewed as indicative of comparatively new to intermediate urban developments with a moderate spatial impact. Hence, the $L_{{cv}_{2}}L_{age}$ cluster is assumed to signify processes of recent and/or on-going suburbanization as well as peri-urbanization. Peri-urbanization could be conceptualized as rapid, fragmented, peripheral urbanization, forming a transitioning zone between rural and urban land [46]. Peri-urban areas are expected to absorb significant amounts of urban growth [47]. The counterpart to $L_{{cv}_{2}}L_{age}$ is formed by the $H_{{cv}_{2}}H_{age}$ cluster, which denotes urban land of moderate to high age in combination with a higher mean *cv*_2_, being significantly higher than that of $L_{{cv}_{2}}L_{age}$ and $L_{{cv}_{2}}H_{age}$ (Table 1). This suggests that $H_{{cv}_{2}}H_{age}$ indicates comparatively large-scale extensions of urban land, e.g., planned city extensions, adjacent to or within urban core areas. This hypothesis is also supported by the cluster’s mean age, which pinpoints to 1960s developments (Fig 6). It may, however, also be seen as indicative of processes of intra-urban redevelopments, redensification, and/or re-urbanization, which are thought to occur predominantly in older urban cores.

### The demographic link: LISA clusters and urban population change

**Fig 7:**
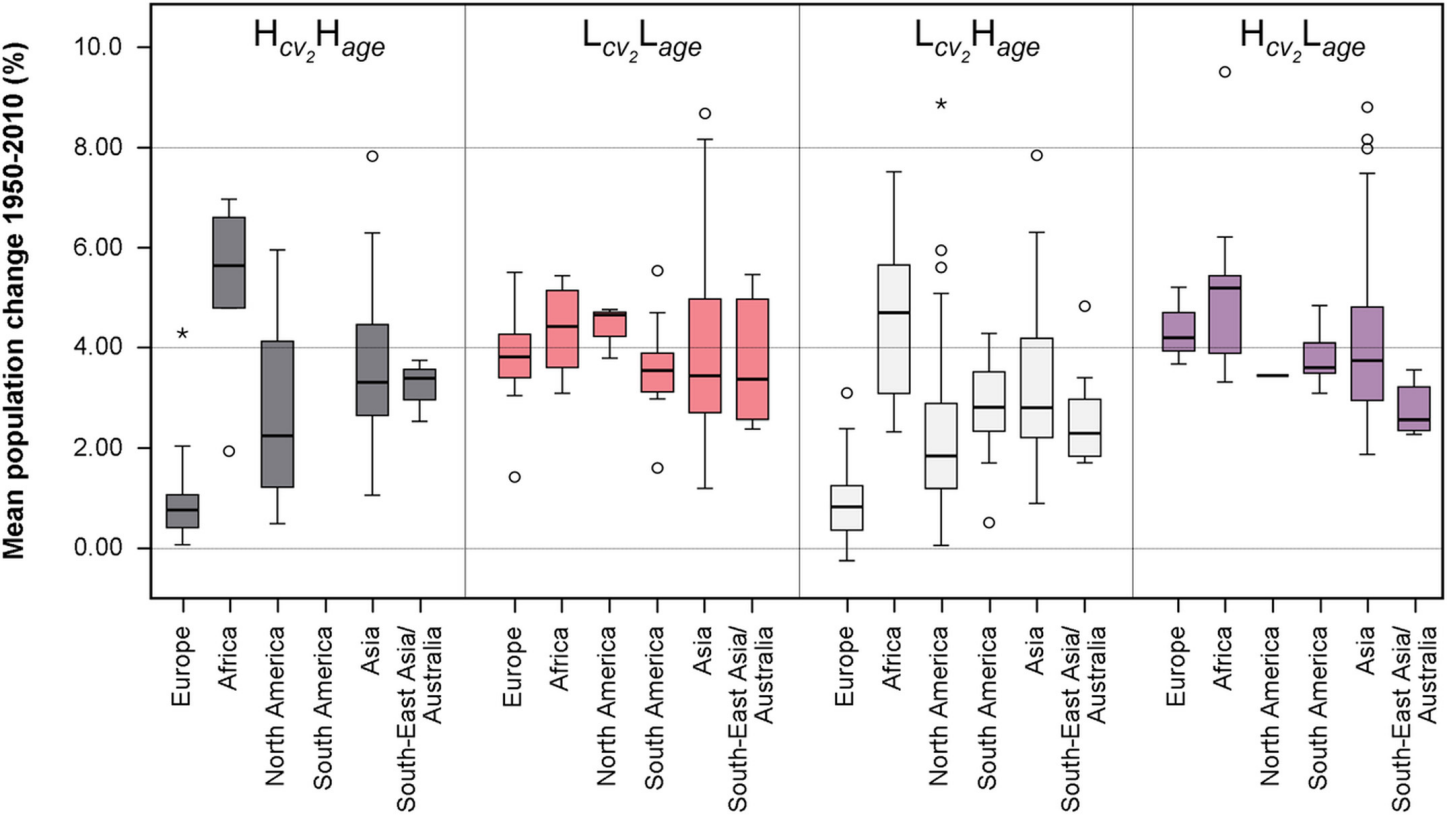
Mean relative total urban population change per LISA cluster and world region. The mean relative change of urban population (%) for the period 1950–2010 has been taken from the World Urbanization Prospects (WUP) dataset [42]. This city-level data has been mapped to grid cells, and thus LISA clusters, and has subsequently been aggregated per world region and LISA cluster: $H_{{cv}_{2}}H_{age}$, High-high cluster; $L_{{cv}_{2}}L_{age}$, Low-low cluster; $L_{{cv}_{2}}H_{age}$, Low-high cluster; $H_{{cv}_{2}}L_{age}$, High-low cluster.

Previously, hotspots of ongoing or recent processes of urbanization have been identified, with the $H_{{cv}_{2}}L_{age},\;L_{{cv}_{2}}L_{age}$, and $H_{{cv}_{2}}H_{age}$ clusters being thought to absorb the majority of urban population growth. This finding is supported by population figures, which have been taken from World Urbanization Prospects (WUP) data [43]. This dataset includes the historical and projected change rates of total urban population for 5-year periods ranging from 1950 to 2030 for the municipality level for various agglomerations with 300,000 or more inhabitants worldwide. The WUP data has been linked to the model's data by mapping the relative population change (in %) for a city to the grid cells covering the area in question, using LandScan-derived data for the delineation of administrative boundaries. This mapping was possible for 418 cases. For these cases, the mean relative population change for the period 1950–2010 has been computed and subsequently been aggregated at two levels: the world region, and the LISA clusters. Fig 7 shows the result of this aggregation.

Examining Fig 7, the mean relative total urban population change rates vary significantly between (i) the different LISA clusters; and (ii) the different world regions. It can be seen that the identified hotspots of urbanization—in particular $H_{{cv}_{2}}L_{age}$—share significantly higher total population change rates compared to $L_{{cv}_{2}}H_{age}$, i.e., urban core cities. No significant difference in the relative population change rate was found between the $H_{{cv}_{2}}L_{age}$ and $L_{{cv}_{2}}L_{age}$ cluster (Table 2). This indicates that population growth is concentrated not only in newly founded urban areas but also in the suburban and peri-urban land; by contrast, population in the core cities is stagnating or growing at much lower rates. These results appear to be in accordance with the findings e.g. for Ho Chi Minh City (VN) and Beijing (CN) [47, 48]. This pattern is also in agreement with the findings of Sánchez-Vidal et al. [34], which state that ‘younger’ cities tend to have higher total population growth rates than ‘older’ ones. It is furthermore in support of findings of Desmet & Rappaport, who study the applicability of Gibrat’s law on US settlements for a period of 200 years [49]. They conclude that (i) younger, and thus smaller settlements, feature higher population growth rates compared to older ones; and (ii) growth rates of young, small settlements are negatively correlated with initial population size, whereas growth rate and population size of intermediate to large—and thus typically older—places are slightly positively correlated and tend to transition towards Gibrat’s law [49].

**Table 2:** Pairwise comparison of LISA cluster mean rank of mean relative total urban population change.

	$H_{{cv}_{2}}H_{age}$	$L_{{cv}_{2}}L_{age}$	$L_{{cv}_{2}}H_{age}$
$L_{{cv}_{2}}L_{age}$	↑		
$L_{{cv}_{2}}H_{age}$	↓	↓	
$H_{{cv}_{2}}L_{age}$	↑	○	↑^a^

$H_{{cv}_{2}}H_{age}$, High-high cluster; $L_{{cv}_{2}}L_{age}$, Low-low cluster; $L_{{cv}_{2}}H_{age}$, Low-high cluster; $H_{{cv}_{2}}L_{age}$, High-low cluster; ↑, mean rank significantly higher; ↓, mean rank significantly lower; ○, no significant difference.

^a^ The table is read line-by-line. For example, the high-low cluster ($H_{{cv}_{2}}L_{age}$) has a significantly higher mean rank of relative total urban population change than the low-high cluster ($L_{{cv}_{2}}H_{age}$).

**Fig 8:**
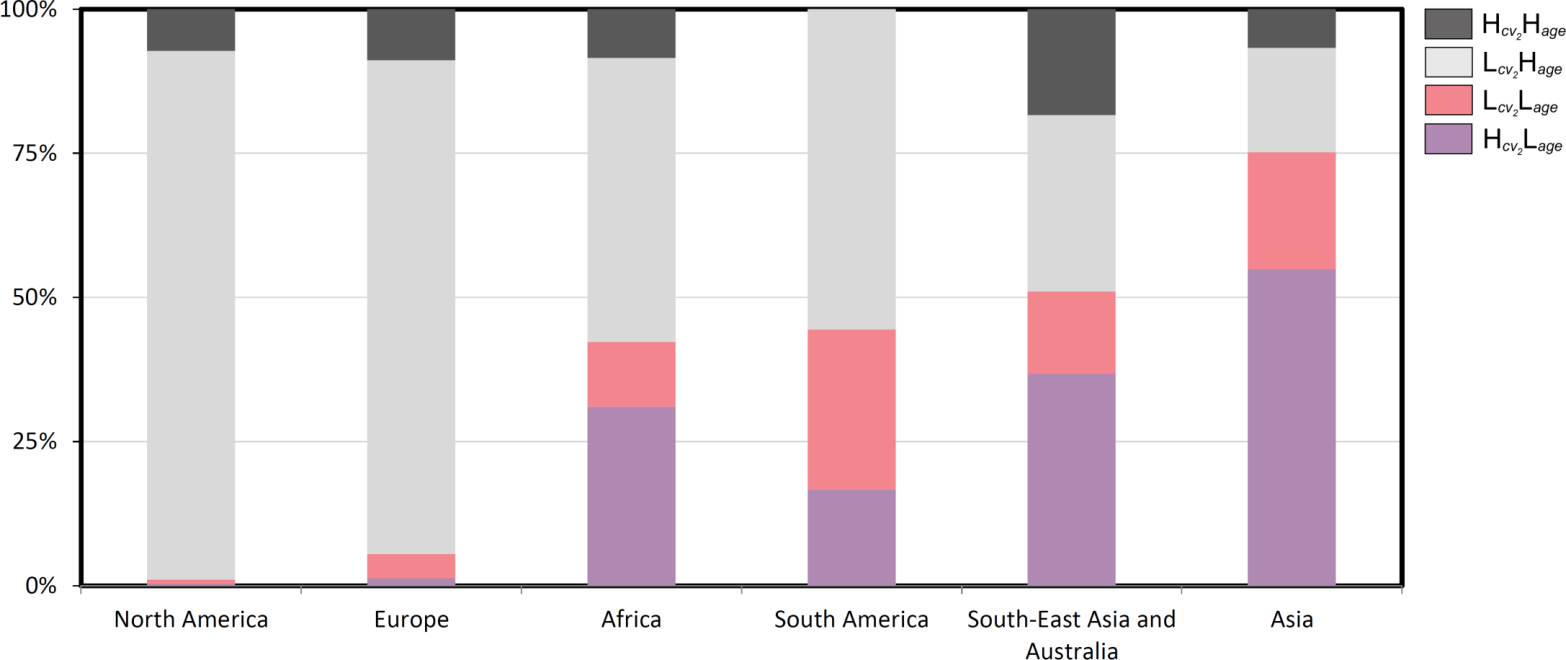
Share of each LISA cluster per world region. The stacked column chart visualizes the (cumulative) share of each LISA cluster per world region: $H_{{cv}_{2}}H_{age}$, High-high cluster; $L_{{cv}_{2}}H_{age}$, Low-high cluster; $L_{{cv}_{2}}L_{age}$, Low-low cluster; $H_{{cv}_{2}}L_{age}$, High-low cluster. In the chart, the regions are sorted by the combined share of $H_{{cv}_{2}}L_{age}$ and $L_{{cv}_{2}}L_{age}$ in ascending order. The $H_{{cv}_{2}}L_{age}$ and $L_{{cv}_{2}}L_{age}$ clusters are seen as absorbing large amounts of urban population growth. Clearly, this combined share is high in Africa, South America, and South-East Asia/Australia, and particularly high in Asia. This corresponds to those world regions for which highest urban population growth rates are projected [2].

When looking at the population change rates per continent it can be found that, for nearly all clusters, the median total population change rate for Africa is, together with Asia and South America, among the highest observed (Fig 7); this supports the findings that view these world regions as hotspots of rapid urban growth [1, 19]. This fact is further reflected by our findings by looking at the share of each LISA cluster per world region. Looking at Fig 8, it becomes clear that the share of urban land classified as $H_{{cv}_{2}}L_{age}$ and $L_{{cv}_{2}}L_{age}$ is 42.3% in Africa, 44.4% in South America and 75% in Asia. In addition, in South-East Asia/Australia, that share is also high, at 51% (Fig 8). These findings confirm the massive urbanization these regions are experiencing and expecting in the future [2]. By contrast, the lowest population change rates have been observed for Europe and North America. The low share of urban hotspots in these regions is in line with this.

### From LISA Clusters to a Conceptual Model of Global Urbanization

**Fig 9:**
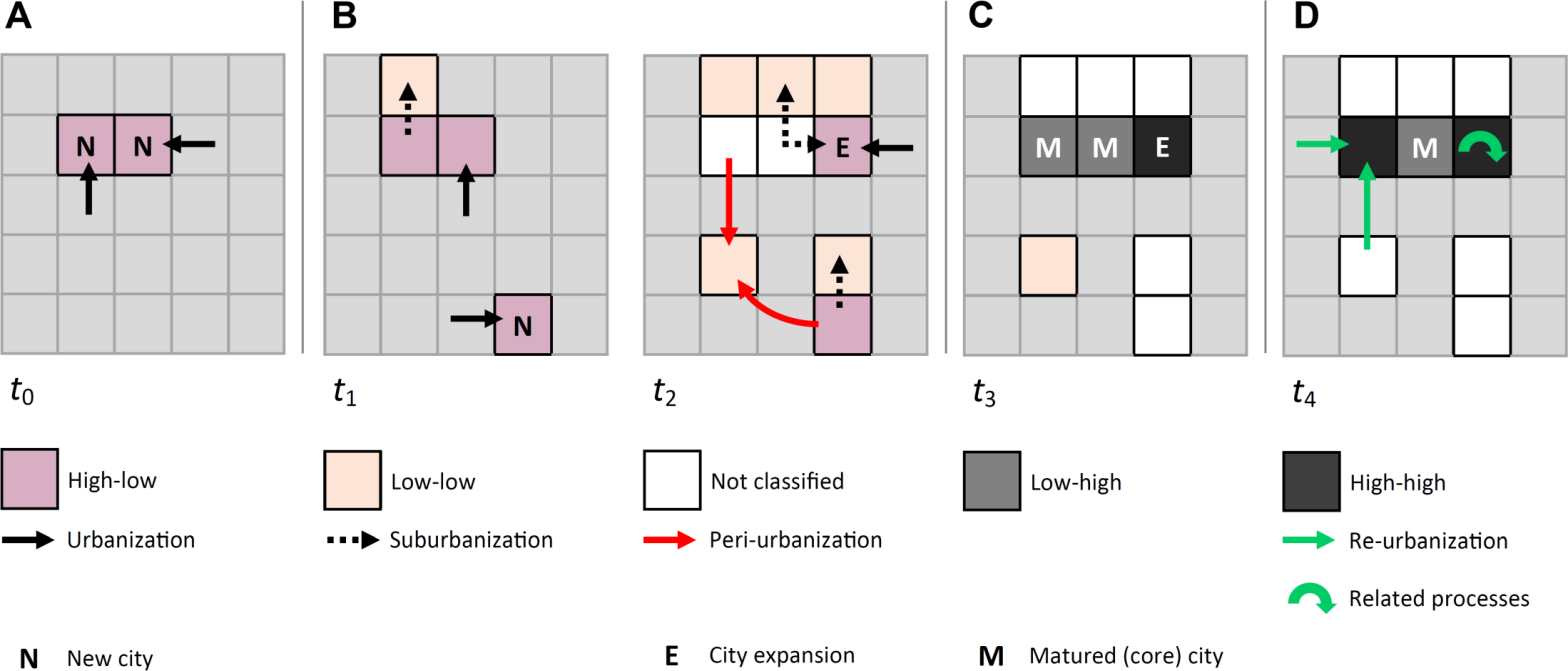
Conceptual model of the spatial relations between the LISA clusters and the corresponding states and processes of urbanization, the latter being shown as arrows. (A) Urbanization (polarization) results in the foundation of a new city (*t*_0_). (B) At a certain point, suburbanization, and thus spread, sets in (*t*_1_). Increasingly more land is transformed into urban land at the urban fringes. Likewise, remote locations in a core city's hinterland are affected by peri-urbanization, leading to the transformation of rural space into rural-urban land (*t*_2_). (C) Large-scale spatial dynamics within the core cities themselves eventually come to a halt, e.g., due to the unavailability of convertible land; therefore, core cities mature. However, large-scale city extensions may be erected to absorb further demographic pressure (*t*_3_). (D) Finally, re-urbanization may set in (*t*_4_).

In the previous section, four LISA clusters have been identified, and we have hypothesized on their conceptual meaning. We have argued that the four clusters might serve as anchor points of urban development, which in turn has been described by polarization and spread. We have assumed that polarization is captured by the clusters of negative spatial autocorrelation. Here, the $H_{{cv}_{2}}L_{age}$ cluster is seen as a “starting point” of this development, since it is thought to signify ongoing, recent urbanization. The $L_{{cv}_{2}}H_{age}$ cluster is seen as representative of the "end point" of such polarization, i.e., as being characteristic of emerged "mature" core cities. Furthermore, we have argued that the clusters of positive spatial autocorrelation may be considered as reference points characterizing urban spread [16], i.e., processes of urbanization occurring at the fringes and within the periphery of urban cores. In this context, the $L_{{cv}_{2}}L_{age}$ cluster has been seen as potentially representative of suburbanization and peri-urbanization. The $H_{{cv}_{2}}L_{age}$ cluster is assumed to represent large-scale dynamics, e.g., planned city extensions such as the “grand ensembles”. It has also been seen to likely indicate processes of redevelopments, redensification, and re-urbanization. Building upon these findings, a spatially explicit conceptual model shall be devised (Fig 9).

As shown in Fig 9, the proposed model seeks to trace the development of a hypothetical urban region from a very early stage of urbanization (*t*_0_) to a more pronounced urban network (*t*_4_), similar to the core-periphery model of urban development by Friedmann [20]. The different, underlying processes of urbanization, each of which resulting in a particular spatial impact mostly in form of the transformation of rural into urban land, are captured in the model in a schematic manner. This includes the stages of urbanization and suburbanization, as devised e.g. by van den Berg, as well as transformation of the rural hinterland into a peri-urban space [15, 20]. As shown in Fig 9D, the model also seeks to capture re-urbanization. It is worthy to note that re-urbanization may be accompanied by additional processes occurring in older urban cores such as intra-urban redevelopments or redensification. Whilst not yet studied in more detail, the model envisages linkages to such potentially spatially explicit related processes (Fig 9D). Hence, the proposed model—despite schematic in nature at this point—is sought to provide a theoretical spatial context needed to trace spatially explicit urban development over time and should thus be regarded as a building block for future research.

## Discussion and Conclusions

In this paper, we have presented an analysis of the relationship of the age of urban area and the spatial dynamics of built-up land for a full coverage of the world. It could be seen that there are strong links between both variables. We have argued that the process of urbanization as a whole can be captured using both variables, which seem to mark one dimension of that process that we have referred to as the spatiotemporal dimension. This spatiotemporal dimension has in turn been described by two axis or pathways of urban development as results of a bivariate Local Moran’s I analysis. A first development path connecting so-called clusters of negative spatial autocorrelation has been conceptualized as capturing processes of polarization leading to the emergence of urban cores. In particular, the high-low cluster is assumed to highlight hotspots of urbanization in terms of a rapid transformation of land into urban, built-up spaces. A second axis connecting clusters of positive spatial autocorrelation is thought to be representative of urban spread. We have argued that the low-low cluster captures processes of suburbanization and peri-urbanization, an increasingly important component of the urbanization process, whilst the high-high cluster might be indicative of large-scale city extensions, as well as re-urbanization and related processes, e.g., redensification. Together, these three clusters may be seen as most relevant when it comes to quantify and qualify the spatial impacts of urbanization as a whole. We have also shown that linking urban population figures to our dataset generally supports the hypothesized “meaning” of LISA clusters. In particular, it could be seen that the postulated hotspots of urban development have indeed amongst the highest urban population growth rates. It could further be shown that this finding, put into context with the share of each LISA cluster per world region, can indeed explain the high growth of urban population that is expected in the Global South [1,2,19]. On the global scale of the analysis, our results thus reflect and confirm findings in the literature.

Contrary to these hotspots of urbanization, we have argued that a fourth cluster, the low-high cluster, is highlighting urban land that is relatively stable in its spatial extent, which is thought to be representative of matured core cities. This cluster might be seen as an “end point” of urban development. Especially for Europe, findings indicate a stagnating or declining urban population within these cores, and we have also found comparatively low growth rates for this cluster in North America. Again, these findings are in line with various studies [18, 50], which highlight shrinkage as another facet of urban development that needs to be taken into account.

It is in this context where limitations of the presented approach arise. In its current form, i.e., without additional data, the model is incapable to distinguish between urban growth and urban shrinkage. This is mostly due to the often very local effects of urban shrinkage [51]. Generally, we have argued that a high value for *cv*_2_ corresponds to comparatively large changes in urban area extent, in relation to the previously present urban land. It follows that these changes may be the result of both urban growth and shrinkage. Moreover, shrinkage is most likely expected in old urban areas. Consequently, it could be assumed that the high-high cluster might additionally capture these effects of shrinkage. Since, in each grid cell, we look at the urban space as a whole, local changes to urban area as a result of shrinkage might however be masked by concurrent changes to urban extent as a result of growth. Considering the global scale of the analysis, it is assumed that the latter effect outnumbers the former. This effect may further be exacerbated by the resolution of the underlying HYDE 3.1 dataset, which in itself has been discussed as a source of potentially high uncertainties—especially in connection to earlier time steps—in regard to the allocation and estimation of the extent of built-up land. Furthermore, the scaling of the coefficient of variation, *cv*_2_, hampers the distinction of growth and shrinkage, since it can only assume positive values. Hence, future analysis should include additional data, e.g., absolute urban area or population, to distinguish between growth and shrinkage and to properly capture effects of shrinkage.

Finally, also the mapping of urban population figures needs to be considered in this regard. In particular, it needs to be noted that the mean relative population change rate used for the analysis does not allow to differentiate between coexisting areas of population growth and decline within a city or agglomeration, neither in an empirical nor spatially explicit manner. Moreover, it is clear that the presented allocation of urban population change rates is relatively coarse. This, however, lies in the very small-scale nature of the global analysis. Another limitation stems from the classification of urban areas by means of bivariate Local Moran's I, which relies upon statistical significance. As indicated in Fig 9, such significance is obviously not always achieved. More precisely, lack of statistical significance is the result of differences in the variables *cv*_2_ and/or age between two grid cells being too small. This results in a possible lack of classification of grid cells, i.e., the respective urban land, into LISA clusters. However, a lacking significance does not mean that observed differences in *cv*_2_ and/or age are non-existent. Hence, we believe that an interpretation of both values, and their “patterns” respectively, is sufficient to draw first important conclusions.

Despite these various uncertainties, the proposed model is considered to show the potential of capturing the urbanization process in a spatial manner for a global scale. The conceptualized LISA cluster meanings—urbanization, suburbanization, peri-urbanization, re-urbanization and potentially shrinkage—also confirm the cyclic character of urban development devised by van den Berg [15] in a non-spatial manner. Future work will need to verify the assumptions made further and in more detail, thereby also validating the conceptual model and developing it further. The presented work is nonetheless seen as a large step forward towards compensating for the lack of spatial context that arises from missing geo-coding of (socio-demographic) data on urban areas, particularly in developing countries [52], that exhibit rapid growth and thus are the urban hotspots of the future [5, 53]. It is in those countries that stakeholders most urgently need a spatially explicit view to manage urban growth and all its related effects to improve the quality of life for billions of people. It is here where we see the proposed model as a suitable means to highlight hotspots for action to be taken.

## Supporting Information

S1 Fig Visualization of variable coefficients of the Varimax-rotated solution (cf. S2 Table). (TIF)

S1 Table Percentage of grid cells that follow a “chronological order” of observations—i.e., an assumption of “spatiotemporal continuity”—over the analysed time steps. For each number of observations of urban land, the time steps are listed for which built-up land is expected to be observed in a given grid cell if a “chronological order” of urban development holds true. It becomes clear that if such is the case, the observation count can be used to deduce the age of built-up land. The percentage of class total gives the share of grid cells per age class which follow that assumption. The percentage of total cases gives the share of class total of all N = 76630 cases. E.g., 94.40% of all grid cells with an observation count of 2 have also been observed in the expected time steps, 1980 and 2000. These grid cells correspond to 26.3% of all cases. Please note that the total cases do not sum up to 100%. The difference, 8.5%, corresponds to the share of total grid cells not following the expected “chronological order”.
(DOCX)

S2 Table Eigenvalues and total variance explained (%) per component. The extracted components with an eigenvalue greater than one are highlighted.
(DOCX)

S3 Table Factor loadings of each variable on the extracted components with an eigenvalue greater than one. The table shows that the variables cv, cv_1_, cv_2_ and age load on component 1, the variables total urban population count and total urban area load on component 2. Coefficients < 0.4 have been suppressed in the table output to ease interpretation.
(DOCX)
