# Supplementary figures and images for: On the Nexus of the Spatial Dynamics of Global Urbanization and the Age of the City
Source: PLoS One. 2016 Aug 4;11(8):e0160471. doi: 10.1371/journal.pone.0160471 (PMC4973923; doi:10.1371/journal.pone.0160471)

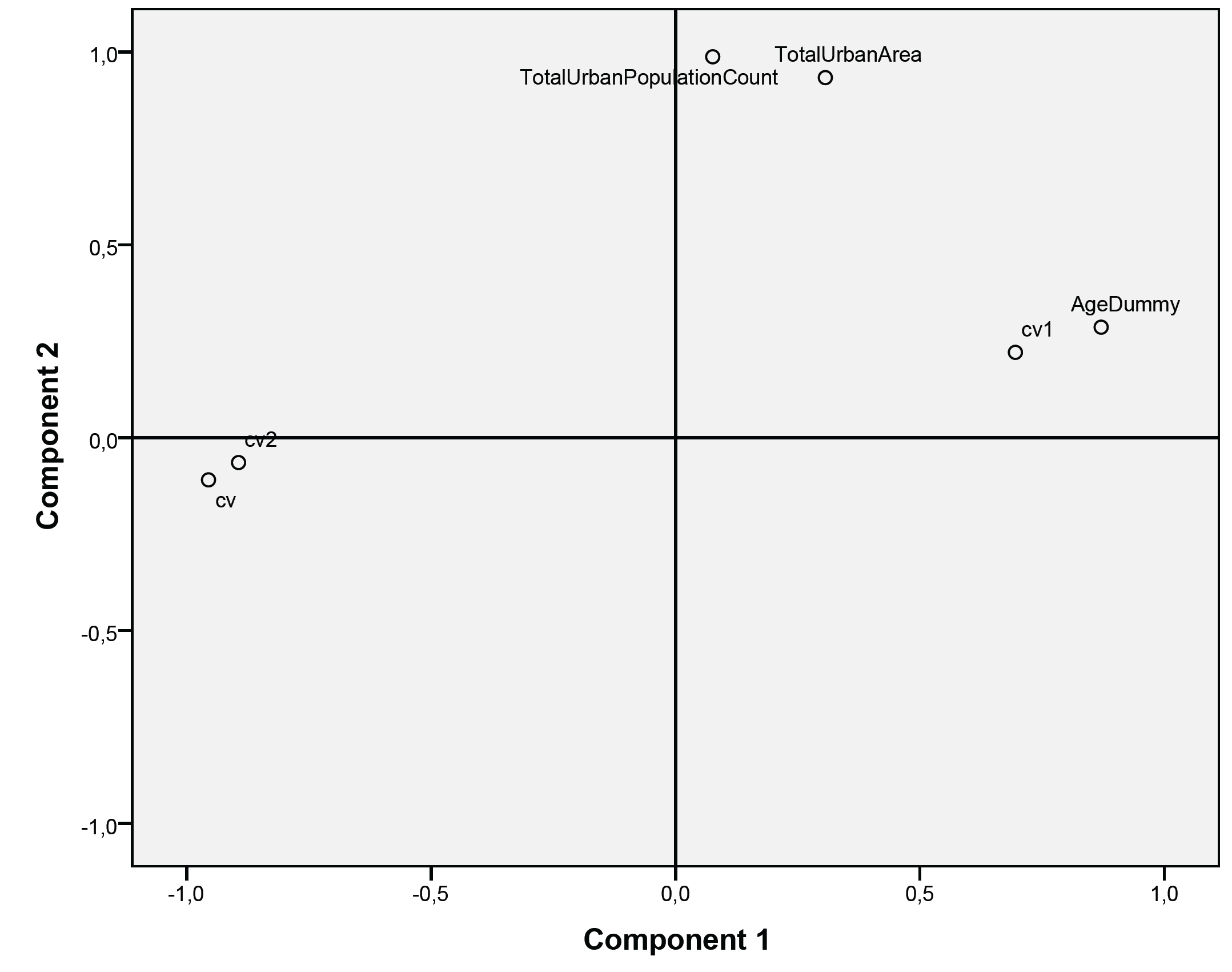

Supplement: S1 Fig — (TIF) [file pone.0160471.s001.tif]
