# Supplementary material for: On the Nexus of the Spatial Dynamics of Global Urbanization and the Age of the City
Source: PLoS One. 2016 Aug 4;11(8):e0160471. doi: 10.1371/journal.pone.0160471 (PMC4973923; doi:10.1371/journal.pone.0160471)
Supplement: S1 Table — For each number of observations of urban land, the time steps are listed for which built-up land is expected to be observed in a given grid cell if a “chronological order” of urban development holds true. It becomes clear that if such is the case, the observation count can be used to deduce the age of built-up land. The percentage of class total gives the share of grid cells per age class which follow that assumption. The percentage of total cases gives the share of class total of all N = 76630 cases. E.g., 94.40% of all grid cells with an observation count of 2 have also been observed in the expected time steps, 1980 and 2000. These grid cells correspond to 26.3% of all cases. Please note that the total cases do not sum up to 100%. The difference, 8.5%, corresponds to the share of total grid cells not following the expected “chronological order”. (DOCX) [file pone.0160471.s002.docx]

**S1 Table. Percentage of grid cells that follow a “chronological order” of observations—i.e., an assumption of “spatiotemporal continuity”—over the analysed time steps.** For each number of observations of urban land, the time steps are listed for which built-up land is expected to be observed in a given grid cell if a “chronological order” of urban development holds true. It becomes clear that if such is the case, the observation count can be used to deduce the age of built-up land. The percentage of class total gives the share of grid cells per age class which follow that assumption. The percentage of total cases gives the share of class total of all N=76630 cases. E.g., 94.40% of all grid cells with an observation count of 2 have also been observed in the expected time steps, 1980 and 2000. These grid cells correspond to 26.3% of all cases. Please note that the total cases do not sum up to 100%. The difference, 8.5%, corresponds to the share of total grid cells not following the expected “chronological order”.

| Number of observations of urban land | Expected time steps of observations if “chronological order” of observations holds true | Percentage of cases following the expected observations | |
| --- | --- | --- | --- |
| (age class) |  | % of  class total | % of  total cases |
| 1 | 2000 | 92.7 | 27.7 |
| 2 | 1980, 2000 | 94.4 | 26.3 |
| 3 | 1960, 1980, 2000 | 85.1 | 8.9 |
| 4 | 1950, 1960, 1980, 2000 | 73.4 | 4.3 |
| 5 | 1940, 1950, 1960, 1980, 2000 | 80.4 | 5.1 |
| 6 | 1920, 1940, 1950, 1960, 1980, 2000 | 88.1 | 3.7 |
| 7 | 1900, 1920, 1940, 1950, 1960, 1980, 2000 | 100.0 | 15.5 |
