# Supplementary material for: On the Nexus of the Spatial Dynamics of Global Urbanization and the Age of the City
Source: PLoS One. 2016 Aug 4;11(8):e0160471. doi: 10.1371/journal.pone.0160471 (PMC4973923; doi:10.1371/journal.pone.0160471)
Supplement: S2 Table — The extracted components with an eigenvalue greater than one are highlighted. (DOCX) [file pone.0160471.s003.docx]

**S2 Table. Eigenvalues and total variance explained (%) per component.** The extracted components with an eigenvalue greater than one are highlighted.

| Component | Initial Eigenvalues | | | Extraction Sums of Squared Loadings | | | Rotation Sums of Squared Loadings | | |
| --- | --- | --- | --- | --- | --- | --- | --- | --- | --- |
|  | Total | % of Variance | Cumulative % | Total | % of Variance | Cumulative % | Total | % of Variance | Cumulative % |
| 1 | 3.590 | 59.833 | 59.833 | 3.590 | 59.833 | 59.833 | 3.052 | 50.873 | 50.873 |
| 2 | 1.456 | 24.262 | 84.094 | 1.456 | 24.262 | 84.094 | 1.993 | 33.222 | 84.094 |
| 3 | 0.600 | 10.001 | 94.095 |  |  |  |  |  |  |
| 4 | 0.247 | 4.118 | 98.213 |  |  |  |  |  |  |
| 5 | 0.067 | 1.119 | 99.332 |  |  |  |  |  |  |
| 6 | 0.040 | 0.668 | 100.000 |  |  |  |  |  |  |
