# Supplementary material for: On the Nexus of the Spatial Dynamics of Global Urbanization and the Age of the City
Source: PLoS One. 2016 Aug 4;11(8):e0160471. doi: 10.1371/journal.pone.0160471 (PMC4973923; doi:10.1371/journal.pone.0160471)
Supplement: S3 Table — The table shows that the variables cv, cv1, cv2 and age load on component 1, the variables total urban population count and total urban area load on component 2. Coefficients < 0.4 have been suppressed in the table output to ease interpretation. (DOCX) [file pone.0160471.s004.docx]

**S3 Table. Factor loadings of each variable on the extracted components with an eigenvalue greater than one.** The table shows that the variables cv, cv_1_, cv_2_ and age load on component 1, the variables total urban population count and total urban area load on component 2. Coefficients < 0.4 have been suppressed in the table output to ease interpretation.

| Variable | Component | |
| --- | --- | --- |
|  | 1 | 2 |
| *cv* (%) | -0.955 |  |
| *cv*_2_ (%) | -0.894 |  |
| *age* | 0.871 |  |
| *cv*_1_ (%) | 0.695 |  |
| Total urban population count |  | 0.987 |
| Total urban area (m²) |  | 0.933 |
